# Neural Network of Body Representation Differs between Transsexuals and Cissexuals

**DOI:** 10.1371/journal.pone.0085914

**Published:** 2014-01-20

**Authors:** Chia-Shu Lin, Hsiao-Lun Ku, Hsiang-Tai Chao, Pei-Chi Tu, Cheng-Ta Li, Chou-Ming Cheng, Tung-Ping Su, Ying-Chiao Lee, Jen-Chuen Hsieh

**Affiliations:** 1 Department of Dentistry, School of Dentistry, National Yang-Ming University, Taipei, Taiwan; 2 Integrated Brain Research Unit, Department of Medical Research, Taipei Veterans General Hospital, Taipei, Taiwan; 3 Department of Psychiatry, Taipei Veterans General Hospital, Taipei, Taiwan; 4 Department of Psychiatry, Shuang Ho Hospital, Taipei Medical University, Taipei, Taiwan; 5 Department of Obstetrics and Gynecology, Taipei Veterans General Hospital, Taipei, Taiwan; 6 Department of Obstetrics and Gynecology, Faculty of Medicine, School of Medicine, National Yang-Ming University, Taipei, Taiwan; 7 Department of Medical Research, Taipei Veterans General Hospital, Taipei, Taiwan; 8 Department of Psychiatry, Faculty of Medicine, School of Medicine, National Yang-Ming University, Taipei, Taiwan; 9 Institute of Brain Science, National Yang-Ming University, Taipei, Taiwan; University of Udine, Italy

## Abstract

Body image is the internal representation of an individual’s own physical appearance. Individuals with gender identity disorder (GID), commonly referred to as transsexuals (TXs), are unable to form a satisfactory body image due to the dissonance between their biological sex and gender identity. We reasoned that changes in the resting-state functional connectivity (rsFC) network would neurologically reflect such experiential incongruence in TXs. Using graph theory-based network analysis, we investigated the regional changes of the degree centrality of the rsFC network. The degree centrality is an index of the functional importance of a node in a neural network. We hypothesized that three key regions of the body representation network, i.e., the primary somatosensory cortex, the superior parietal lobule and the insula, would show a higher degree centrality in TXs. Twenty-three pre-treatment TXs (11 male-to-female and 12 female-to-male TXs) as one psychosocial group and 23 age-matched healthy cissexual control subjects (CISs, 11 males and 12 females) were recruited. Resting-state functional magnetic resonance imaging was performed, and binarized rsFC networks were constructed. The TXs demonstrated a significantly higher degree centrality in the bilateral superior parietal lobule and the primary somatosensory cortex. In addition, the connectivity between the right insula and the bilateral primary somatosensory cortices was negatively correlated with the selfness rating of their desired genders. These data indicate that the key components of body representation manifest in TXs as critical function hubs in the rsFC network. The negative association may imply a coping mechanism that dissociates bodily emotion from body image. The changes in the functional connectome may serve as representational markers for the dysphoric bodily self of TXs.

## Introduction

Individuals with gender identity disorder (GID) [1], commonly referred to as transsexuals (TXs), protractedly suffer from an incongruence between their identified genders and physical sexes or bodies [1]. As opposed to TXs, cissexual (CIS) people feel the sexual anatomy they were born with is right for them (mental genders and physical sexes are and always have been aligned). We previously reported that the TXs as compared to the CISs, demonstrate an increased functional connectivity between the ventral tegmental area and anterior cingulate cortex subregions that signatures the psychosocial distress for the gender-sex incongruity of TXs [2]. Such distress can be substantially ascribed to a disharmonic body image (an internal representation of an individual’s own physical appearance) and a dysmorphic body experience [3], i.e., the dissatisfaction of his or her own physical appearance. In fact, TXs may achieve better health after sex reassignment surgery due to the increased satisfaction with one’s own body [4]–[6]. Therefore, a different body image as compared to that of CIS people may be one key psychological attribute of TXs. However, the neural mechanisms underpinning the body image processing in TXs have not yet been explored.

The neural network of body representation has been suggested to serve three major functions: somatosensation, somatoperception and somatorepresentation [7]. Somatosensation, the processes of encoding primary sensory somatic stimuli, is associated with the primary somatosensory cortex at the postcentral gyrus (PostC) [8]. Somatoperception, the processes of constructing the percepts and experiences of one’s own body, is associated with the superior parietal lobe (SPL) [7]. Somatorepresentation, the process of constructing body-related knowledge and attitudes, relates to the circuitry of semantic knowledge and is predominantly associated with the left frontal and parietal lobes [7]. The somatosensory cortex has strong structural and functional connections with the insula (IC) [9], [10] (a neural substrate crucial for the overall somatorepresentation), which integrates interoceptive, exteroceptive and emotional information and contributes to the awareness of body status [11], [12]. The IC is also a critical component of the salience network, and together with the anterior cingulate cortex (ACC), unites conflict monitoring, interoceptive-autonomic and reward-processing [13].

The resting-state functional connectivity (rsFC) during low-frequency oscillations, as studied by functional magnetic resonance imaging (fMRI), may reflect the brain state of self-referential internal representation [14] and exteroceptive and interoceptive deployment of attention [15]. The rsFC brain network, as a form of system memory [16], can be sculpted by long-term experiences, including complex learning of art [17], chronic stress and traumatic experiences [18], and psychosocial distress for the gender-sex incongruity of TXs [2]. We employed the graph theory-based network analysis, quantifying the topological features of the rsFC network, composed of nodes (parcellated brain regions) and edges (inter-nodal FC), to elucidate the neural architecture of parallel information processing among regions (nodes) of brain [17], [19], [20]. A functional hub in the context of neural network is pivotal for high-level cognitive functions because it coordinates the overall information flow and maintains the integrity of the brain connectome [20], [21]. The degree centrality (DC), a measure of local network connectivity, was specifically used to index the functional level of a hub in the neural network [20], where an increased DC of a node indicates a greater functional role in inter-regional communication and integration [19].

It has been shown that the participants with grapheme-color synesthesia, compared with the otherwise normal controls, revealed an increased DC in the structural network of the brain [22]. Both brief sessions of motor training and transcranial direct current stimuli applied over the primary somatosensory area result in an increased network centrality in the motor-related network [23], [24]. The consistent findings of experience- and learning-based plasticity in the brain pinpoint that sustained experience and intensive learning could be coupled with a regional increase of DC [22]–[24] and associated with a long-lasting increase of intrinsic connectivity strength or a change of intrinsic connectivity pattern in the pertinent brain regions [16], [24]. This is also evidenced by our previous seed-based functional connectivity study on TXs [2]. TXs have both aversive feeling to their incongruent body parts and the heightened aversive emotions associated with the sensation of them. These psychophysical elements are further compounded by the transsexual behavior and gestures. Therefore, we hypothesized that TXs as compared to CISs can be associated with changes in the aforementioned neural network for the overall body representation. Such changes can be manifested with an increase of DC in the PostC, SPL and IC, as well as with different intrinsic connectivity patterns linking TX’s subjective experience.

## Materials and Methods

### Participants

Individuals who met the diagnostic criteria of GID, commonly referred to as transsexuals (TXs), herein, according to the Diagnostic and Statistical Manual of Mental Disorders, Fourth Edition Text-revised (DSM-IV-TR) [1], were recruited from the psychiatric clinic of the Taipei Veterans General Hospital (TXs, n = 23) from 2010 to 2012. Age-matched healthy cissexual control subjects (CISs, n = 23, 11 males and 12 females) were recruited from electronic bulletin board systems via an Internet advertisement (see Table 1 of [2] for the participant demographic profiles). All subjects (TXs and CISs) had participated in our previous behavioral and neuroimaging study of psychosocial distress of TXs [2]. All TXs had neither received hormone treatment nor sex reassignment surgery and were without any neurological or major psychiatric disorders (see Table 1 for detailed inclusion and exclusion criteria). TXs consisted of 13 female-to-male transsexuals (FTMs) and 10 male-to-female transsexuals (MTFs). MTFs are biological males who identify themselves as female and have the desire to be female, and FTMs are biological females who identify themselves as male and have the desire to be male. Females and FTMs were neither in the ovulatory period nor pregnant during the study period. Participants abstained from sexual behavior the day before the experiment. All participants were assessed using a visual analogue scale (0 = *none*, and 10 = *the maximum imaginable*) regarding the level of their *self-identification as the opposite sex* (IOS) and the *desire to become the opposite sex* (DOS). The TXs demonstrated a significantly higher level of IOS and DOS relative to the CISs (please refer to our previous report [2]). The IOS and the DOS assessments were used as an accessory measurement for gender dysphoria in clinical evaluation. Neither the IOS nor DOS was used in the subsequent analyses. We were mainly interested in comparing the TXs as one psychosocial group (the MTFs and FTMs combined) to the CISs as one group (cissexual males and females combined). This rationale was based on a previous report demonstrating that no differences were observed between the MTFs and FTMs in pertinent psychological and behavioral assessments (for detailed clinical, psychological and behavioral measurements, please refer to our previous report) [2]. This study was approved by the institutional review board of the Taipei Veterans General Hospital. Written informed consents were given by all participants.

**Table 1:** Inclusion and exclusion criteria for the TX and the CIS groups.

*Inclusion criteria for the CIS group*	
1.	Written informed consent approved by the *institutional review board* (IRB)
2.	Aged 20–40 years old
3.	Sexual orientation according to the Klein Sexual Orientation Grid: Average score of A to G <4 and total scores <56
***Inclusion criteria for the TX group***	
1.	After a clinical psychiatric interview according to the Diagnostic and Statistical Manual of Mental Disorders (DSM-IV-TR), transsexuals met the criteria for gender identity disorder (GID) and no other major psychiatric disorders. They had not received sexual reassignment surgery.
2.	Written informed consent approved by the *institutional review board* (IRB)
3.	Aged 20–40 years old
4.	Homosexual sexual orientation according to the Klein Sexual Orientation Grid: Average score of A to G >4 and total scores >56
***Exclusion criteria (for both the TX and the CIS groups)***	
1.	Visual problems (except those corrected by glasses)
2.	Current or previous physical or neurological diseases
3.	Current medical treatments
4.	Meet the diagnosis of other psychiatric disorders (except for GID in the TX group)
5.	Arizona Sexual Experience Scale (ASES) total score >18 or score of any item >5
6.	With experience watching erotic films and those with feelings of aversive disgust against the film scenes most of the time (more than half) while watching the erotic films (It is the “disgust against the scenes”, not the “disgust about their bodies”).
7.	A history of sexual abuse
8.	Females in their ovulatory period*
9.	Sexual contact leading to orgasm 24 hours before the study
10.	The consumption of alcohol, tea or coffee 24 hours before the study
11.	Pregnancy
12.	Not applicable for MRI study

Subjects who were in the period ranging from less than 11 days (follicular phase) or more than 17 days after the beginning of their last menses were included. Follow-up phone calls were made to verify the date of the beginning of the next menses. This selection criterion was used on the basis that sudden surges in LH (luteinizing hormone) and FSH (follicle stimulating hormone) at mid-menstrual cycle could affect brain activation patterns.

### Psychological Assessment

The Beck Depression Index (BDI) [25] was used to assess the participants’ moods. The validated Chinese version of the Defense Style Questionnaire (DSQ) [26], [27] was used to assess their psychosocial coping strategies. The sexual orientations of the TXs and CISs were assessed by the Klein Sexual Orientation Grid [28]. The details of the psychological assessments were provided in our previous work [2].

### Behavioral Study

The participants were subjected to a behavioral study [see [2] for details]. In brief, they watched four silent erotic (E) and four silent neutral (N) films (30 s each) in a balanced, semi-randomized order. The E-films contained scenes of male-female genital intercourse in the nude, whereas the N-films contained scenes of common male-female dialogue in regular clothing. Immediately after each film, the participants rated their erotic arousal *(arousal* score) according to the statement, “*Please rate the degree to which you felt sexually aroused when watching this film,*” and the extent to which they felt embodied as male or female (*selfness* score) according to the statement, “*Please rate the degree to which you identify yourself as the male or female in the film.*” Participants rated directions of both genders respectively. For the psychological ratings, the participants used a visual analogue scale (0 = *none*, and 10 = *the maximum imaginable*). The selfness scores were used in the following correlation analysis for the intrinsic network connectivity.

### Acquisition of Resting-state Functional Imaging Data

Resting-state fMRI images were obtained using a 3.0-T MRI scanner (Discovery MR750, GE Inc., USA) at the Taipei Veterans General Hospital. Gradient echo EPI (Echo Planar Imaging) is used for fMRI scanning. The scanning parameters were as follows: gradient echo T2* weighted sequence, [TR]/[TE] = 2000 ms/30 ms, [FOV] = 230×230 mm^2^, matrix size = 64×64, 40 slices/image volume, slice thickness = 4 mm and 205 volumes per run. The initial five scans were discarded due to signal saturation. During scanning, the participants were instructed to remain awake with eyes open and fixated on a cross symbol on the projection screen and to be relaxed. The participant is requested to press the balloon after the fMRI is finished. High-resolution T1 structural images were acquired in the sagittal plane using a high-resolution sequence ([TR]/[TE] = 8.208 ms/3.248 ms, [FOV] = 230×230×158.4 mm^3^ and matrix = 256×256×176). The 3D-MPRAGE is adapted with inversion RF pulse to enhance T1 contrast in 3T. TI (Inversion Time) is equal to 450ms in this study. These structural images are acquired in axial plane. The total time of imaging experiment was approximately seven minutes. The scanning was performed within two weeks after the behavior study. The delay should be ascribed to the limited research-time availability on the clinical scanner as used in the current study.

### Preprocessing of the Resting-state Functional Imaging Data

Preprocessing was performed using SPM8 software (Statistic Parametric Mapping, http://www.fil.ion.ucl.ac.uk/spm). All scans were slice timing-corrected, head movement-corrected and normalized to the Montreal Neurological Institute (MNI) template. Because head motion has a significant systematic effect on rsFC MRI network measures [29], we excluded participants with head motion of any volume >2 mm or 2° from our samples; one male CIS participant was therefore excluded. Spurious or nonspecific sources of variance were removed by the regression of the following variables: (a) the six movement parameters computed based on rigid body translation and rotation in preprocessing, (b) the mean signal within the lateral ventricles, and (c) the mean signal within the deep white matter (defined by thresholding the SPM5’s *a priori* white matter mask (white.nii) at 90% [30]). We did not perform global signal regression due to the recent debate on the potential distortion of correlation patterns [31], [32]. The resulting time series was band-pass filtered (0.01–0.1 Hz) to extract the low-frequency oscillating components that contributed to rsFC.

### Construction of the Graph theory-based Connectivity Network

In this study, we applied graph theory-based network analysis to investigate the architecture of the brain connectivity networks [19]. This method has been widely used for investigating how the rsFC network is sculpted by long-term experiences, such as complex learning [17] and mental disorders [21]. This model of the rsFC network is constructed based on a graphical approach composed of nodes (i.e., parcellated brain regions, see Table 2 for all neuroanatomical abbreviations) and edges (i.e., rs-connectivity between regions). A graph is composed of nodes and edges (i.e., inter-nodal links). We defined the nodes as the brain regions of the rsFC network and the edges as the inter-nodal rsFC connectivity (see below).

**Table 2:** Abbreviations for the anatomical regions defined by the Harvard-Oxford cortical and subcortical atlases.

Abbreviation	Anatomical Region
Fpole	Frontal Pole
IC	Insular Cortex
SFG	Superior Frontal Gyrus
MFG	Middle Frontal Gyrus
IFGtriang	Inferior Frontal Gyrus, pars triangularis
IFGoper	Inferior Frontal Gyrus, pars opercularis
PreC	Precentral Gyrus
Tpole	Temporal Pole
STGant	Anterior Superior Temporal Gyrus
STGpost.	Posterior Superior Temporal Gyrus
MTGant	Anterior Middle Temporal Gyrus
MTGpost	Posterior Middle Temporal Gyrus
MTGto	Temporoccipital part of Middle Temporal Gyrus
ITGant	Anterior Inferior Temporal Gyrus
ITGpost	Posterior Inferior Temporal Gyrus
ITGto	Temporoccipital part of Inferior Temporal Gyrus
PostC	Postcentral Gyrus
SPL	Superior Parietal Lobule
SMGant	Anterior Supramarginal Gyrus
SMGpost	Posterior Supramarginal Gyrus
ANG	Angular Gyrus
LOCsup	Superior Lateral Occipital Cortex
LOCinf	Inferior Lateral Occipital Cortex
IntraCAL	Intracalcarine Cortex
FMC	Frontal Medial Cortex
SMA	Juxtapositional Lobule*
Subcallosal	Subcallosal Cortex
ParaCG	Paracingulate Cortex
ACG	Anterior Cingulate Gyrus
PCG	Posterior Cingulate Gyrus
PCUN	Precuneus Cortex
CUN	Cuneal Cortex
FOrb	Frontal Orbital Cortex
PHIPant	Anterior Parahippocampus
PHIPpost	Posterior Parahippocampus
LIN	Lingual Gyrus
TFUSant	Anterior Division of Temporal Fusiform Cortex
TFUSpost	Posterior Division of Temporal Fusiform Cortex
TOFus	Temporal Occipital Fusiform Cortex
OFus	Occipital Fusiform Cortex
FOper	Frontal Operculum
COper	Central Operculum
POper	Parietal Operculum
PPolare	Planum Polare
HES	Heschl’s Gyrus
PTemporale	Planum Temporale
SupraCAL	Supracalcarine Cortex
Opole	Occipital Pole
THA	Thalamus
PUT	Putamen
CAU	Caudate
PAL	Pallidum
HIP	Hippocampus
AMYG	Amygdala
NAC	Nucleus Accumbens

Formerly termed the Supplementary Motor Cortex.

#### Definition of the network nodes

The network nodes were defined as the parcellated brain regions. Parcellation was performed according to the Harvard-Oxford cortical and subcortical probabilistic atlases from FSLView v3.1 (http://fsl.fmrib.ox.ac.uk/fsl/fslview) using a 25% threshold. The atlases consist of bilateral 48 cortical and seven subcortical structural areas (Table 2). For each node (region), the regional mean time series were calculated by averaging the time series of each voxel of the corresponding node. The bilateral PostC, SPL and IC were specifically assigned as the nodes of interest (NOIs) due to their role in the body representation circuitry. The PostC region is bordered rostrally by the central sulcus and caudally by the postcentral sulcus, corresponding to the cytoarchitectonically defined primary somatosensory cortex [33].

#### Definition of the network edges

The network edge was defined based on the Pearson correlation coefficient (r) between the regional mean time series of each node pair. The resulting r was then converted to a z score using Fisher’s r-to-z transformation to improve the normality. The absolute value of the z score was indexed as the weight of an edge. To create networks with a different density, for each participant, the z score matrix was thresholded at different density levels using the minimum spanning tree (MST) method [34], with two different (global and local) thresholds. Most brain network analyses applied only global thresholding (T) for the construction of a connectivity network. Only if the functional connectivity (i.e., z score) of a pair of brain regions exceeds the given threshold T, an edge is deemed to exist [34]. Nevertheless, the network could be disrupted and fragmented with emergence of isolated islands (singular nodes) when a low global threshold is set to create a low-density or sparse network. The existence of isolated island(s) is neuroscientifically unexplainable and unjustified. To solve this problem, the MST method had been introduced to facilitate group comparisons of networks by forcing the connectedness of sparse graphs [17]. In brief, for each node, the edge with the highest weight was retained to instantiate the contact with at least one neighboring node. This step (termed as *local thresholding*) was iteratively performed for every node in the brain to warrant that every node (region) in the network (brain) were in connection with one another. The local thresholding procedure thus gives rise to a fully connected *backbone* network. Subsequently, we applied a global threshold and selected edges between the pairs of nodes with the highest functional connectivity in order [34]. For technical details, please refer to [34] and [17]. We investigated the network constructed over a range of network density values, ranging from 1% to 40%, at an increment of 1%. For each network density, a binary network was created by setting edge weights less than the threshold as 0 and edge weights greater than the threshold as 1.

### Statistical Analyses

#### Quantitative changes in the degree centrality

We calculated the DC of each node, which quantified the importance of a node as a functional hub in the brain network [20]. All of the following analyses were performed on a binary network for which the DC is defined as the number of the edges connecting a node. To test our first hypothesis regarding the distinct body representation network in TXs, we directly compared the DC for each NOI (i.e., the bilateral PostC, SPL and IC) between the TX and the CIS groups using two-sample independent t-tests (TX>CIS, with an alpha value = 0.05). The comparison was performed across a range of low network densities (1–12%). This range was selected because only the stronger edges (i.e., inter-nodal connectivity) were preserved at low density.

#### Qualitative changes in the connectional pattern

We further investigated the nodes that connected with the NOIs. The analysis was performed at the lowest network density that showed a significant difference in the DC (i.e., 3%; see *Results*). For each node, we averaged the value of its edges connected with a specific NOI for all participants. This NOI-specific edge mean value (i.e., *group connectivity*) represents the inter-subject consistency of connection between the node and the NOI. A high mean value indicates that a connection between the node and the NOI exists in the majority of the participants.

#### Regression analysis

To address the mind-brain correspondence in the neural somatorepresentation of TXs, we investigated the correlation between the selfness rating vs. the connectivity between the IC and PostC and the connectivity between the IC and SPL, respectively. The selfness scores denoted the degree to which the participants focused their identification with the desired genders (i.e., the gender with which TXs participants cross-identify) and un-desired/dis-identified genders (i.e., the gender that TXs participants feel discomfort with or the original sex). The IC-PostC and IC-SPL connectivity were regressed with the selfness scores.

#### Investigation of methodological variations

For the rsFC network analysis, variations in methodology (e.g., analysis of a binary or weighted network and with or without the intermediate step of co-registration to T1 images in the image processing) may result in different outcomes [35]. Therefore, we validated our observations using results obtained from different methodological approaches. We also compared the DCs of each NOI between groups at a network density of 3% using the following configurations: (a) an additional analysis using the weighted-network approach; and (b) an analysis using functional images obtained from a procedure that includes the preprocessing of T1 co-registration.

## Results

### Quantitative Changes in the Degree Centrality

**Figure 1:**
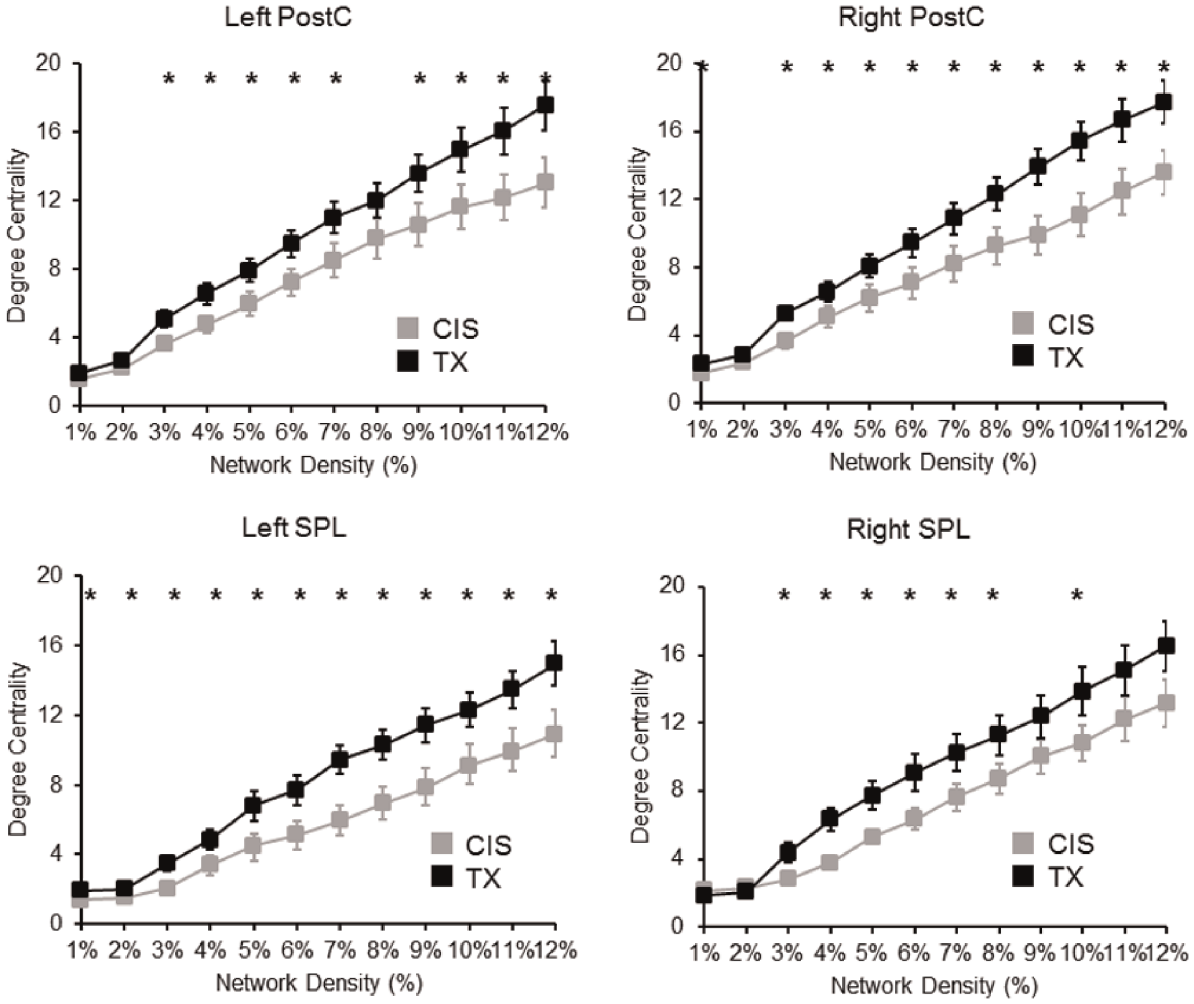
The between-group difference in the degree centrality of the node of interests (NOIs). All NOIs showed an increased degree centrality (one-tailed two-sample t-test, TX>CIS) across a range of network densities (1–12%). An asterisk denotes p<0.05.

**Figure 2:**
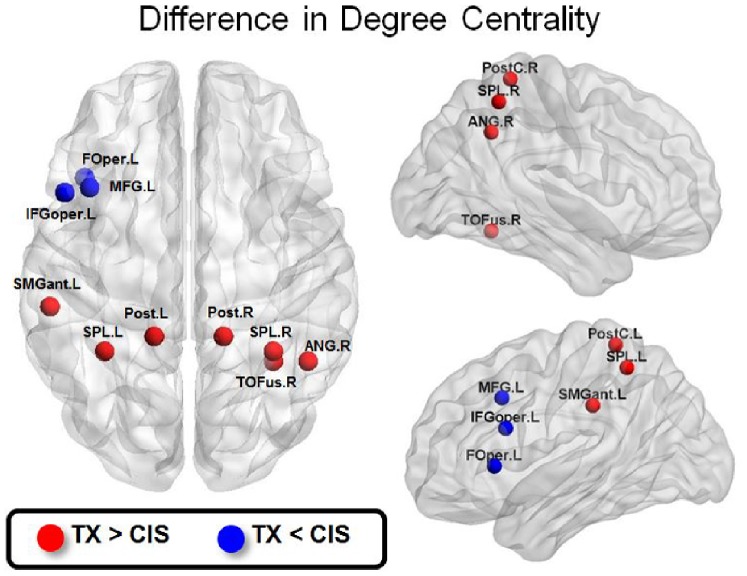
The between-group difference in the degree centrality of all nodes. Only the nodes with significantly increased (red) or decreased (blue) degree centrality in the TX group vs. the CIS group are shown (one-tailed two-sample t-test, alpha = 0.05). The network density is 3%.

Both the bilateral PostC and SPL showed a significantly increased DC in the TX group compared with the CIS group. The changes in the DC were significant over a range of network densities (1–12%) (Figure 1). These findings supported our first hypothesis regarding a different body representation network of TXs. However, we did not observe an increased DC at the bilateral ICs in the TX group compared to the CIS group. Additionally, the right temporal occipital fusiform cortex (TOFus) showed a significantly increased DC, and the left inferior frontal gyrus (the opercular part, IFGoper), the left MFG and the left FOper showed a significantly decreased DC over a range of network densities (Figure 2).

### Qualitative Changes in the Connectional Pattern

**Figure 3:**
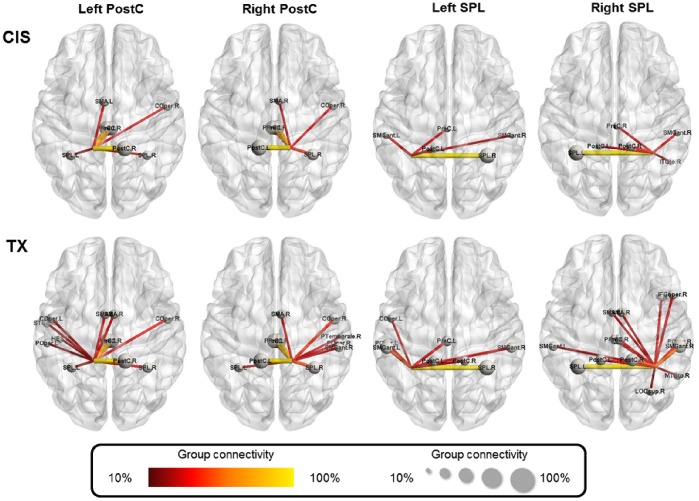
Connectional pattern of the node of interests (NOIs). The nodes with stronger connections (i.e., group connectivity >10%) within each of the NOIs are displayed for the TX and the CIS groups. The nodal size and edge color denote the strength of the group connectivity between a node and the NOI. Stronger group connectivity indicates that a larger number of participants shared the same edge in their binary networks.

When comparing the TX group with the CIS group, the PostC and SPL showed a different pattern of inter-nodal connectivity with the other nodes. In the TXs, the PostC connected with the temporal lobe nodes (STGant, SMGant, HES and PTemporale), whereas the SPL additionally connected with the occipital lobe nodes (LOCsup), the sensory area (POper) and the motor area (SMA) (Figure 3).

### Regression Analysis

**Figure 4:**
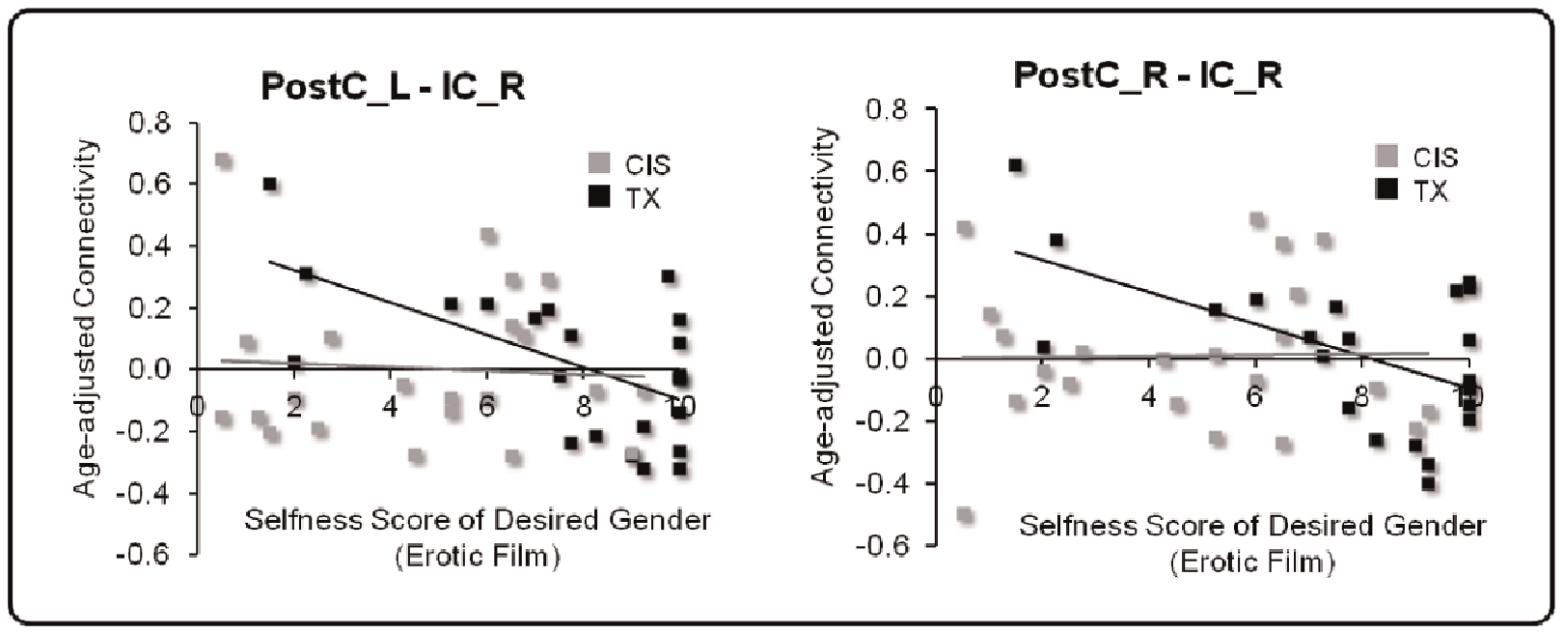
Regression analysis. The connectivity (i.e., the weight of the edge and z score) between the right Post and IC as well as the left Post and right IC are plotted against the selfness score that the TX participants rated for the characters with their identified genders. In the TX group, the connectivity showed a significant negative correlation with the selfness score (right PostC: *r* = −.57, *p*<.005; left PostC: *r* = −.60, *p*<.005).

In the TX group, the connectivity between the right IC and the right and left PostC was negatively correlated with the selfness score of the desired genders (right PostC: r = −.57, p<.005; left PostC: r = −.60, p<.005). After analyzing the CIS group, no such significant correlation was found (Figure 4). In both groups, no significant correlation was observed between the selfness score of the undesired genders and connectivity. The findings affirmed the role of the IC in the body image processing of the TX group. The connectivity between the left IC and the bilateral PostC did not show a significant correlation with the selfness score in both groups.

### Investigation of Methodological Variations

The application of varied approaches revealed that neither the constitution property of the network (binary or weighted) nor the co-registration with the T1 image for the image processing influenced the FC changes. For each NOI, the TX group consistently showed a DC higher than that of the CIS group (see Text S1, Figures S1 and S2).

## Discussion

### Distinct Body Representation Network in TXs

We demonstrated that the bilateral PostC and SPL, two key components of the body representation circuitry, are functionally more expressed in terms of a high DC as hubs in TXs (Figures 1 and 2). Qualitatively, the regions featured a different pattern of connections with other nodes in the rsFC network. In the TX group, the body representation network showed a more extensive connection with the brain regions associated with sensorimotor processing (Figure 3). These findings suggest a distinct neural circuitry of the body representation in TXs, which is in agreement with their long-term dysphoric experience with their physical bodies. These findings are corroborated by evidence that the MTFs showed structural (GM volume) changes in the pre/postcentral gyrus and the thalamus, which are related to the processing of body perception [36]. Collectively, these convergent findings posit a neurological base for the dysphoric experience of TXs [37].

The brain regions associated with visual processing and face recognition (i.e., the LOC and TPOFus) also demonstrated an increased DC in the TX group (Figure 2). Furthermore, the TX group showed a greater number of connections between the PostC and SPL and the brain regions associated with visual processing (Figure 3, the ‘LOC’ node) and auditory processing (Figure 3, the ‘PTemporale’ node). In contrast, the body presentation network of the CIS group is more confined within the parietal lobe (Figure 3). Both auditory (e.g., the voice) and visual (e.g., body appearance) experiences are critical attributes for body image and gender identification [4]. Therefore, the constellations of the LOC, TPOFus and PTemporale indicate that the TXs substantially incorporate visual and auditory cues for the central sculpturing of their body image. A recent fMRI study using independent component analysis revealed that anorexia nervosa patients (a disorder where the disturbance of body image is the main characteristic) showed a significantly increased co-activation between the somatosensory network and the superior parietal cortex [38]. Another study on the structural connectivity of patients with body dysphoric disorder reported an increased node centrality within the occipital lobe and the temporal pole [39]. Our findings suggest that the neural substrates servicing the somatic experience and processing of information from other sensory attributes collectively constitute the neural ensemble of body image representation for TXs. Notably, TXs also demonstrated a greater number of connections between the PostC and SPL and the SMA (Figure 3). The SMA in the Harvard-Oxford cortical probabilistic atlas includes the posterior pre-SMA and SMA proper. Together they subserve versatile functions, including intention, preparation, anticipation, ideation, and fine motor control [40], [41]. Both the SMA and the parietal cortices, including the SPL, are important components of the sensorimotor network for the bodily self [42]. Our data suggest that the TX body representation system additionally engage the SMA for the overall sentient self.

Additionally, the TX-associated changes in topological features may reflect changes in body-related attention. The SPL plays a key role in maintaining sensorimotor integration and updating information about the current body state [43]. Anticipation of somatosensory stimuli would lead to biased attention to certain body part(s) [44] that engages the primary somatosensory cortex [45]. Therefore, in the TX group the increased DC in the PostC and the SPL may reflect an altered/heightened attention to their incongruent body part(s).

### Role of the Insula in Shaping Body Image

The IC is critical for the awareness of body status [11], [12]. As a prominent component of the salience network, the IC unites conflict monitoring, interoceptive-autonomic and reward-processing [13]. We did not observe significant DC changes in the IC or ACC of TXs. An extensive change in insular function is associated with mental illness, such as schizophrenia [46].

In contrast, the connectivity between the right IC and the bilateral PostC in the TX group showed a negative correlation with the selfness score while watching the erotic films. The selfness score reflects the degree that the individuals converge their identification with the character of their desired gender. This finding highlights a greater expression of functional coupling between neural substrates for body representation and body awareness in the TX group. Structurally and functionally, the IC is densely connected with the somatosensory cortex [9], [10]. As a key component of the salience network, the IC is critical for integrating the information of different sensory modalities into subjective awareness of the body to form a subjective body representation [47]. The IC also plays a key role in the emotional processing of somatic stimuli [7]. Moreover, attention to aversive emotion is associated with a co-activation of the right IC and somatosensory cortex [48]. The right IC activation is also related to the “gut feelings” evoked by observing body distortions [49]. Therefore, the inverse correlation between the IC and PostC connectivity and the selfness score of desired gender in the TX group may implicate an individual difference in dissociating the bodily emotion and physical body. A weaker IC-PostC connectivity strength corresponds to a greater extent to which TXs could intentionally disengage their bodily emotion from the actual perceived body state, which in turn can lead to a different level of cross-gender identification. On the other hand, the IC also plays a key role in salience detection and error awareness [47], [50]. The right IC in particular drives the salience network after the detection of incongruence errors [51]. Therefore, the negative correlation between the IC and PostC connectivity and the selfness score of desired gender may be alternatively interpreted as a reduced salience towards the incongruent bodily experience. Active dissociation between the bodily experience and salience as a means to disintegrate the aversive body parts may be a critical coping strategy for TXs to resolve the incongruence between a desired and perceived body image.

### Decreased DC in the Lateral Prefrontal Cortex

We found that the brain regions of the lateral prefrontal cortex (LPFC), including the left MFG, IFGoper and FOper, showed a significantly decreased DC in the TX group vs. the CIS group (Figure 2). In people with early-life stress, the severity of self-reported stress is correlated negatively with global connectivity and the hubness of the left dorsolateral PFC [52]. Major depressives showing a higher level of apathy, as compared to non-apathetic ones, may demonstrate significantly lower functional connectivity between the dorsal ACC and dorsolateral/ventrolateral PFC [53]. Since many TX participants are stressed and even depressed by the dysphoric experience, it is highly possible that the decreased DC in the LPFC may reflect a stress-related disconnection of the LPFC. We had additionally investigated the correlation between the BDI scores and DCs for each node of the left LPFC (i.e., the left middle frontal gyrus [MFG_L], the left inferior frontal gyrus, opercularis [IFGoper_L], and the left frontal operculum [FOper]). None of these nodes show significant correlation with the BDI score (MFG_L: r = −0.22, p>0.05; IFGoper_L: r = −0.18, p>0.05; FOper_L: r = 0.20, p>0.05). Thus the negative findings argue against the depressed mood to be of substantial and causal contribution for the decreased DC of LPFC.

The LPFC plays a key role in the integration of multimodal sensory information and the cognitive regulation of emotion [54], [55]. For example, when viewing erotic films, the participants showed greater activation of the left IFG when they voluntarily self-regulated the intensity of the sexual arousal [56]. Furthermore, the left IFG plays a key role in the processing of inner speech, a self-reflective activity associated with self-regulation and introspection on the emotional response [57]. We speculate that the decreased DC in this region might be indicative of a disengagement or suppression of overt cognitive processing of the emotional self as a coping mechanism for the gender-sex incongruence. This argument is supported by the observation that the TX group demonstrated a higher degree of maladaptive defensive styles for stress coping [e.g., displacement and undoing that was revealed by the DSQ. [See [2]].

### Points of Consideration

In this current study, we parcellated the brain according to an anatomical scheme and defined the area of the postcentral gyrus and superior parietal lobe as the major anatomical components of the body representation circuitry. It can be argued that such anatomically defined regions cannot be fully equated to *functional* regions. It should be noted that the entire postcentral gyrus was used in performing the connectivity analyses. We consider the current approach to be justified because transsexuality involve the masculine and feminine appearances entailing the genital organs, breasts, voices, faces, and whole body shapes. Furthermore, TXs’ distinct neural network of body representation can be coterminal to genetic constitution, developmental factors and learned experience in their life. To our knowledge, there is currently no similar rs-connectivity study, using graph-theory approach, addressing specifically the body representation network in body representation disorders. Thus, it remains elusive whether the connectivity pattern found here is specific to transsexuality. However, given the observations that different mind states as the results of long-term associative learning and experience can be coupled with different brain connectivity patterns [17], we speculate that different forms of body representation disorders or dysphoria may have different connectivity representations.

## Conclusions

Using graph theory-based network analyses, we report that TXs are associated with changes in the resting-state functional connectivity. The DC increases in the body representation network of TXs. The instantiation of functional connectome changes may serve as representational neuro-markers for the subjective experience and distinct body image of TXs.

## Supporting Information

Figure S1 **The between-group difference in the degree centrality, results with weighted networks.**
(TIF)

Figure S2 **The between-group difference in the degree centrality, results with T1-coregsitrated functional images.**
(TIF)

Text S1 (DOCX)
