# Supplementary material for: Neural Network of Body Representation Differs between Transsexuals and Cissexuals
Source: PLoS One. 2014 Jan 20;9(1):e85914. doi: 10.1371/journal.pone.0085914 (PMC3896415; doi:10.1371/journal.pone.0085914)
Supplement: Text S1 — (DOCX) [file pone.0085914.s003.docx]

**Supporting Text S1**

**Investigation of Methodological Variations**

We had compared the DCs (degree centrality) between the TX and CIS groups obtained with different methodological configurations: (1) weighted networks were used instead of binary networks, and (2) T1 co-registered functional images were used instead of non-co-registered images. In general, across the studied range of network densities (1-12%), the TX group showed a higher DC compared with the CIS group in each of the NOIs (right and left PostC and SPL) (Figures S1 and S2). This finding suggests that the nature of the network (weighted vs. binary) and the quality of imaging pre-processing (co-registered or not) would not influence our conclusion regarding the altered body representation network in the TX group.

**Supporting Figure Legends**

**Supporting Figure S1. The between-group difference in the degree centrality, results with weighted networks.** All NOIs showed an increased degree centrality (one-tailed two-sample t-test, TX > CIS) across a range of network densities (1-12%). An asterisk denotes p < 0.05.

**Supporting Figure S2. The between-group difference in the degree centrality, results with T1-coregsitrated functional images.** All NOIs showed an increased degree centrality (one-tailed two-sample t-test, TX > CIS) across a range of network densities (1-12%). An asterisk denotes p < 0.05.
